# Preventable hospitalizations from ambulatory care sensitive conditions in nursing homes: evidence from Switzerland

**DOI:** 10.1007/s00038-019-01294-1

**Published:** 2019-09-03

**Authors:** Ulrike Muench, Michael Simon, Raphaëlle-Ashley Guerbaai, Carlo De Pietro, Andreas Zeller, Reto W. Kressig, Franziska Zúñiga, For the INTERCARE Research Group

**Affiliations:** 1 Department of Social and Behavioural Sciences, University of California San Francisco, School of Nursing, San Francisco, USA; 2 Department of Public Health, Institute of Nursing Science, Faculty of Medicine, University of Basel, Bernoullistr. 28, 4056 Basel, Switzerland; 3 Nursing and Midwifery Research Unit, Inselspital Bern University Hospital, Bern, Switzerland; 4 Department of Business Economics, Health and Social Care at the University of Applied Sciences and Arts of Southern Switzerland, Lugano, Switzerland; 5 Faculty of Medicine, University of Basel, Basel, Switzerland; 6 Center for Primary Health Care, University of Basel, Basel, Switzerland; 7 FELIX PLATTER, University Medicine of Aging, Basel, Switzerland

**Keywords:** Avoidable hospitalizations, Hospital costs, Nursing homes, Preventable hospitalizations

## Abstract

**Objectives:**

Reducing nursing home hospitalizations for ambulatory care sensitive conditions (ACSC) has been identified as an opportunity to improve patient well-being and reduce costs. The aim of this study was to identify number of hospitalizations for ACSCs for nursing home residents in a Swiss national sample, examine demographic characteristics of nursing home hospitalizations due to ACSCs, and calculate hospital expenses from these hospitalizations.

**Methods:**

Using merged hospital administrative data with payment data based on diagnosis-related groups (DRGs) for the year 2013, we descriptively examined nursing home residents who were 65 years of age or older and were admitted to an acute care hospital.

**Results:**

Approximately 42% of all nursing home admissions were due to ACSCs. Payments to Swiss hospitals for ACSCs can be estimated at between 89 and 105 million Swiss francs in 2013.

**Conclusions:**

A sizable share of hospitalizations for nursing home residents is for ACSCs, and the associated costs are substantial. Programs and policies designed to reduce these potentially avoidable hospitalizations from the nursing home setting could lead to an increased patient well-being and lower costs.

**Electronic supplementary material:**

The online version of this article (10.1007/s00038-019-01294-1) contains supplementary material, which is available to authorized users.

## Introduction

Reducing preventable hospitalizations of nursing home residents is important to the health and well-being of nursing home residents (Ouslander and Maslow 2012; Walsh et al. 2012). Hospitalizations of frail, elderly persons with multiple chronic conditions are often related to loss of functional ability, delirium, cognitive decline, falls, hospital-acquired infections, and discontinuity of medications (Boockvar et al. 2004; Dwyer et al. 2014; Palese et al. 2016; Zisberg et al. 2016). Several estimates show that in the USA and Canada, between 20 and 60% of hospitalizations from nursing homes are considered preventable (Mcandrew et al. 2016; Ouslander et al. 2010; Walker et al. 2009; Walsh et al. 2012). These hospitalizations are not only a major burden to residents but also a substantial financial burden to the health-care delivery system, with the costs of a hospitalization averaging between $8000 and $20,000 in the USA (Axon et al. 2015; Grabowski et al. 2007; Mcandrew et al. 2016; Walsh et al. 2010; Xing et al. 2013).

Preventable—avoidable, inappropriate, or unnecessary—hospitalizations fall into two groups: hospitalizations which could have been avoided with evidence-based, chronic disease management, prevention of acute deteriorations of chronic conditions, or hospitalizations for conditions which could have been treated in the nursing home. Ambulatory care sensitive conditions (ACSCs) are a group of conditions which include pneumonia, chronic obstructive pulmonary disease (COPD), or congestive heart failure (CHF), among others, and are one of the approaches to measure preventability. Research conducted in the USA suggests that this set of conditions can be effectively and safely managed in the primary care setting when early detection and adequate chronic disease management are in place (Mcandrew et al. 2016; Walsh et al. 2012). Identifying ACSCs can serve as an approximation of potentially preventable hospitalizations, although the effective management of these conditions outside the hospital setting depends on a variety of factors. For example, the decision for or against an ED visit or hospitalization is guided by residents’ and families’ preferences, the presence of advance care planning, do-not hospitalize and do-not resuscitate orders, timely availability of qualified staff, availability of diagnostic and therapeutic interventions in the nursing home, and the acuteness and severity of the condition (Ouslander and Maslow 2012; Renom-Guiteras et al. 2014). Finally, reimbursement policies might favor hospitalizations (Ashton 2014).

ACSCs were developed for the primary care setting and have been used for both research and policy analysis (Billings et al. 1993; Purdy et al. 2009; Weissman et al. 1992). Several approaches exist in the literature as to which conditions are considered sensitive to ambulatory care (Ansari et al. 2012; Billings et al. 1993; Millman 1993; Purdy et al. 2009; Weissman et al. 1992). Recently, expert panels in the USA and Canada developed groups of conditions specifically for nursing home residents (Walker et al. 2009; Walsh et al. 2012). While there were some inconsistencies in the groups of ACSCs identified, consensus was reached regarding the inclusion of asthma, cellulitis, CHF, COPD, dehydration, gastroenteritis, diabetes complications, hypertension, pneumonia, urinary tract infection (UTI), seizures, and injuries from falls/fractures. Typically, in case of exacerbation of any of these conditions, many residents would need to be treated in the hospital. However, preventing ACSCs is achieved through effective monitoring of resident symptoms in nursing homes, in combination with an interprofessional approach to discuss treatment goals and procedures, to avoid exacerbations when possible. For example, in case of an acute situation, the resident might remain in the nursing home for palliative care. Similarly, residents with a tentative diagnosis of fracture will go to the ED; however, an effective fall management program might prevent fall-related injuries.

While ACSCs do not capture all the factors which contribute to a decision to hospitalize a resident, identifying ACSCs and understanding resident characteristics that are associated with an ACSC can provide valuable insight into whether nursing home residents are or are not receiving high-quality care. The Center for Medicare and Medicaid (CMS), the Agency for Healthcare Research and Quality (AHRQ), the WHO, and the OECD are using ACSCs hospitalizations as quality indicators (OECD 2015; WHO Regional Office for Europe 2016). Thus, identifying hospitalizations that are potentially preventable can provide valuable information for hospital and nursing home administrators, policy makers, and researchers.

The aim of this paper is threefold: to identify ACSCs hospitalizations in Swiss hospitals that are from nursing homes, examine patient characteristics associated with ACSCs, and calculate hospital payments for ACSCs.

## Methods

### Design, setting, and sample

**Fig. 1:**
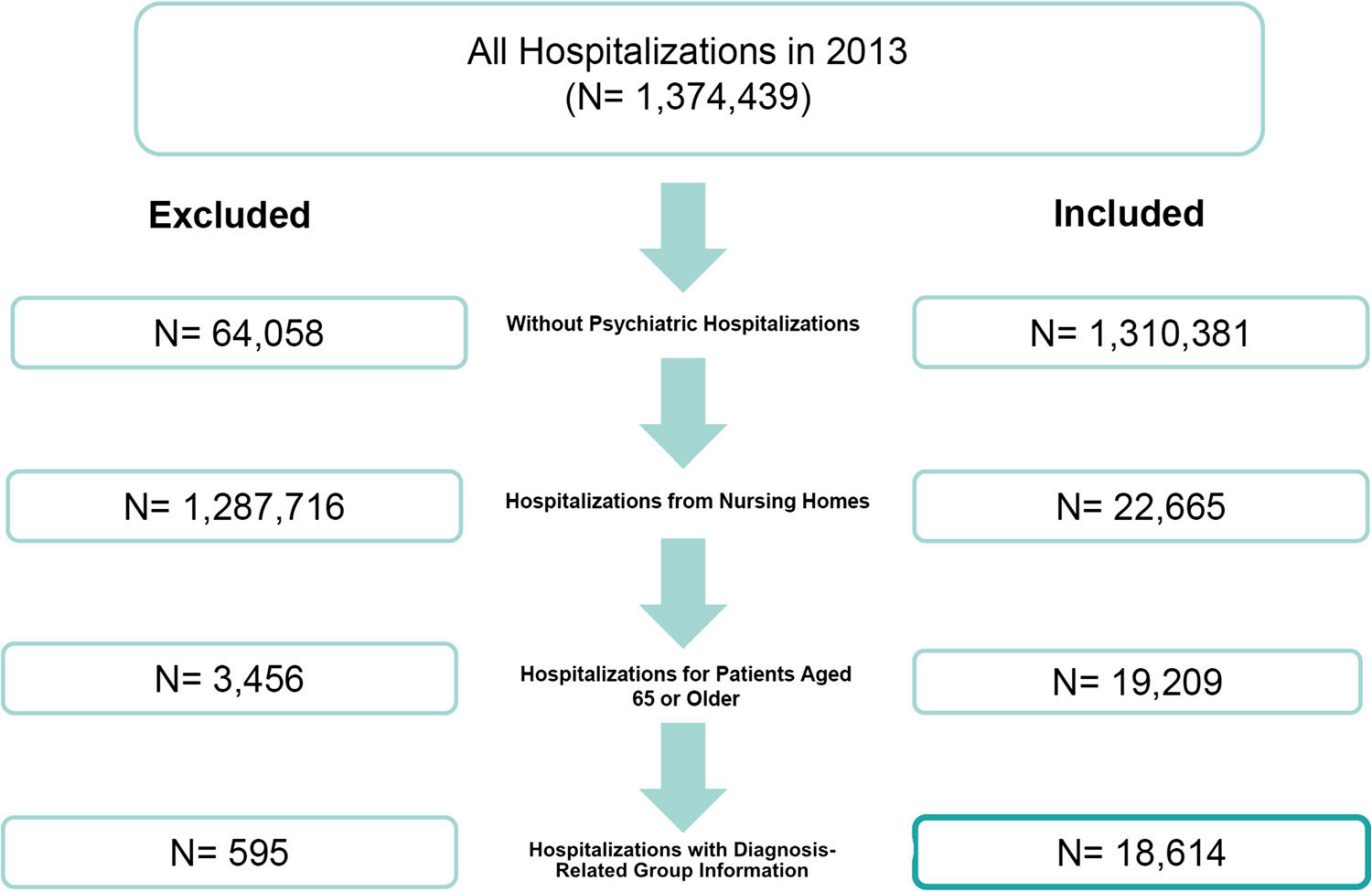
Flowchart of the sample selection process

This study is a retrospective descriptive analysis of nursing home residents’ admissions to hospitals in Switzerland for the year 2013. Our sample consisted of patients aged 65 and older with at least one admission to an acute care hospital. Admissions to psychiatric hospitals were excluded. Figure 1 shows the sample selection process.

### Data

We used data from two different sources: (1) administrative claims from the Medical Statistics of Hospitals (Medizinische Statistik der Krankenhäuser), kept by the Swiss Federal Statistical Office (SFSO), and (2) data on hospital payments for diagnosis-related groups (DRGs) kept by SwissDRG AG (SwissDRG AG 2012). All hospitals in Switzerland are mandated to collect data for every hospitalization and report them yearly to the SFSO (Bundesamt für Statistik). In addition to indicating whether patients had a hospital admission, these data included information on demographics, patient diagnoses, DRGs, length of stay, and discharge location, among other factors. For the purpose of our analysis, we made use of the DRG codes available in both data sets to merge these sources and calculate the costs per admission (more detail below). All data in the Medical Statistics of Hospitals are routinely collected and are de-identified. No ethics approval was necessary.

### Measures

#### Primary outcomes

##### Hospitalizations related to ACSC

We measured the number of hospitalizations for patients who were admitted with ACSCs. ACSCs were identified based on conditions recognized as being sensitive to nursing home care, by the CMS in the USA (Walsh et al. 2010) and by researchers in Canada (Walker et al. 2009). Since the two lists differed, we used conditions which were included in both lists: asthma, poor glycemic control, hypertension, cellulites, dehydration, diarrhea, seizures, UTI, COPD, CHF, pneumonia and bronchitis, falls, and trauma. As these conditions were identified with ICD-9 codes originally, we applied the corresponding Swiss ICD-10 codes [15] (see supplementary material for full list). In case of coding discrepancies (e.g., which codes were attributed to identifying COPD), we followed the ACSC identification outlined by Walsh and colleagues (Walsh et al. 2010).

##### Costs related to ACSCs

Hospital payments in Switzerland are calculated based on cost weights. Cost weights are determined according to the SwissDRG classification system. Each DRG has a cost weight (adjusted annually based on hospital costs). We used this cost weight and multiplied it with a base rate (negotiated between insurers and hospitals). For our study, we used a base rate of 11,200 CHF for university hospitals and 9200 CHF for non-university hospitals, to provide a range of costs attributable to ACSC hospitalizations, because we did not have access to regional information on hospital admissions. This would have allowed us to use more detailed regional base rates for each Canton. In addition, cost weights were adjusted with a predetermined weight for every day a patient’s length of stay fell below or above a certain threshold (SwissDRG AG 2012). For the purpose of our analysis, we did not apply supplementary payments under SwissDRG version 2.0, and did not include supplementary payments for blood transfusions under SwissDRG version 2.0. For a more detailed description of hospital payments, DRGs, and cost weights, see SwissDRG AG (SwissDRG AG). We excluded hospitalizations for which a DRG weight was not available; this was the case for approximately 3% of the sample (see Fig. 1).

### Statistical analysis

Descriptive analyses were performed based on the distribution of the variables (frequencies and percentages, median, and interquartile range).

## Results

### Sample characteristics

Overall, there were 1,374,439 hospitalizations in Swiss hospitals, in 2013, of which 64,058 (4.7%) were to a psychiatric hospital. Of the remaining acute care hospitalizations, 85.6% of patients were admitted from home, 8.8% from other hospitals, 1.7% from nursing homes, and 3.9% from other settings. When focusing on nursing home admissions, there were a total of 19,209 hospitalizations of patients 65 years and older. We combined data from these patients with their available DRG data. A small number of hospitalizations (3%) did not have DRG information that matched with our DRG database. The final analytic sample that met our sample selection criteria outlined in Fig. 1, consisted of 18,614 hospitalizations belonging to 16,215 patients.

Patients were admitted between 1 and 8 times, and 18.1% (3370) of patients had multiple hospitalizations, representing 11% (2002) of the total share of hospitalizations observed in 2013. Table 1 shows the age and gender breakdown for ACSCs versus other conditions. While over half of all hospitalizations were from patients 80 years or older regardless of ACSC status, the shares of hospitalizations for ACSCs were considerably larger for patients 85–90 years of age (26.3% non-ACSC vs. 29.3% ACSC) and for patients 90–95 years of age (15.1% non-ACSC vs. 22.1% ACSC). Women were hospitalized more for ACSCs than for non-ACSCs, while men were less hospitalized for ACSCs.

**Table 1:** Demographic characteristics associated with hospitalizations by ambulatory care sensitive condition status (Swiss Federal Office of Statistics, hospital data 2013), Switzerland 2013

	Non-ACSC (*n *= 10,679)		ACSC (*n *= 7935)		All admissions (*n *= 18,614)	
	*n*	%	*n*	%	*n*	%
Age groups						
65–69	747	7.0	356	4.5	1103	5.9
70–74	1048	9.8	491	6.2	1539	8.3
75–79	1647	15.4	861	10.9	2508	13.5
80–84	2427	22.7	1610	20.3	4037	21.7
85–89	2808	26.3	2324	29.3	5132	27.6
90–94	1615	15.1	1755	22.1	3370	18.1
95+	387	3.6	538	6.8	925	5.0
Gender						
Men	3900	36.5	2354	29.7	6254	33.6
Women	6779	63.5	5581	70.3	12360	66.4

*ACSC* ambulatory care sensitive condition

Table 2 summarizes entry and exit information related to each hospitalization, including mode of admission (self/family, ambulance, physician, other) and the place of discharge. While more residents were admitted for ACSCs than non-ACSCs with an ambulance (41.8% vs. 30.7%), it was the other way round for admissions by a physician (45.7% ACSCs vs. 59.2% non-ACSCs). Approximately two-thirds of hospitalizations were associated with discharges to nursing homes regardless of ACSC status. The share of hospitalizations that were associated with in-hospital mortality was 8.8% for non-ACSCs and 6.9% for ACSCs.

**Table 2:** Entry and discharge information associated with hospitalization by ambulatory care sensitive condition status (Swiss Federal Office of Statistics, 2013), Switzerland 2013

	Non-ACSC (*n *= 10,679)		ACSC (*n *= 7935)	
	*n*	%	*n*	%
Admitted by				
Self or family	610	5.7	569	7.2
Ambulance	3276	30.7	3313	41.8
Physician	6326	59.2	3626	45.7
Other	467	4.4	427	5.4
Place of stay after discharge				
Death	937	8.8	548	6.9
Home	2232	20.9	1188	15.0
Nursing home	6465	60.5	5470	68.9
Other	1045	9.8	729	9.2

*ACSC* ambulatory care sensitive condition

### Potentially avoidable hospitalizations for ambulatory care sensitive conditions

Approximately 42% of hospitalizations from nursing home residents were attributable to an ACSC. The three most common ACSCs were falls and trauma (53.6%), pneumonia and bronchitis (15%), and CHF (12.1%). Pneumonia and bronchitis, CHF, and poor glycemic control were also among the three most common conditions for in-hospital mortality. Table 3 shows the proportions of ACSCs as share of total nursing home hospitalizations by hospital mortality and length of stay.

**Table 3:** Share of ambulatory care sensitive condition hospitalizations by diagnosis group, mortality, and length of stay (Swiss Federal Office of Statistics, 2013), Switzerland 2013

Ambulatory care sensitive condition	Main diagnosis (*n *= 7935)		Mortality (*n *= 548 deaths)		Length of stay (*n *= 7935)	
	*n*	%^a^	*n*	%^b^	Median	IQR
Falls and trauma	4253	22.8	214	5.0	8	8
Pneumonia and bronchitis	1188	6.4	162	13.6	8	6
Congestive heart failure	961	5.2	115	12.0	9	7
Urinary tract infection	402	2.2	8	2.0	7	5
Chronic obstructive pulmonary disease	391	2.1	20	5.1	8	7
Diarrhea and gastroenteritis	285	1.5	6	2.1	7	6
Seizures	207	1.1	12	5.8	6	7
Dehydration	73	0.4	5	6.8	7	5
Hypertension	68	0.4	1	1.5	6	5
Cellulitis	49	0.3	1	2.0	9	9
Asthma	29	0.2	0	0.0	7	5
Poor glycemic control	29	0.2	4	13.8	7	7
Total	7935	42.8	548	6.9		

*IQR* Interquartile range

^a^Percentage in relation to all hospitalizations (*n *= 18,614)

^b^Percentage in relation to each ambulatory care sensitive condition

### Costs associated with avoidable hospitalizations for ambulatory care sensitive conditions

Assuming a base rate for university hospitals of 11,200 CHF and a base rate for non-university hospitals of 9500 CHF, the average cost for an ACSC hospitalization in a university hospital was 13,267 CHF and 11,253 CHF for a non-university hospital, respectively. Costs ranged from 6704/5686 CHF (university/non-university hospitals) for UTIs to 15,332/13,005 CHF (university/non-university hospitals) for falls and trauma. The three most common ACSCs, namely falls and trauma, pneumonia and bronchitis, and CHF, were among the five most expensive hospitalizations. Table 4 shows the average costs per hospitalization for each ACSC.

**Table 4:** Mean admission costs by ambulatory care sensitive conditions (Swiss Federal Office of Statistics, hospital data 2013), Switzerland 2013

	Mean admission costs based on university hospital rate (11,200 CHF)	Mean admission costs based on non-university hospital rate (9500 CHF)
Falls	15,333	13,005
Congestive heart failure	13,013	11,038
Seizures	12,998	11,025
Cellulitis	12,683	10,758
Pneumonia and bronchitis	11,429	9694
Chronic obstructive pulmonary disease	10,768	9133
Dehydration	9589	8134
Poor glycemic control	8735	7409
Hypertension	7651	6490
Asthma	7368	6249
Diarrhea and gastroenteritis	7289	6183
Urinary tract infection	6704	5687

*CHF* Swiss francs

Hospital payments in 2013 for nursing home residents who were admitted with an ACSC totaled roughly 105 million CHF for a university hospital and 89 million CHF for non-university hospitals. In comparison, hospital costs for all conditions for nursing home residents combined were approximately 265 million CHF and 225 million CHF, respectively, for the two different base rates. Thus, the share of the costs associated with ACSCs was approximately 40% of the costs from all conditions. Aligned with the number of hospitalizations observed for each of the ACSCs, falls and trauma, pneumonia and bronchitis, and CHF were responsible for the highest hospital costs. Table 5 provides an overview of the costs for each ACSC and all other conditions for both university and non-university hospital base rates.

**Table 5:** Hospital expenditures for ambulatory care sensitive conditions and all conditions (Swiss Federal Office of Statistics, hospital data 2013), Switzerland 2013

	Expenditures based on university hospital rate (11,200 CHF)	Expenditures based on non-university hospital rate (9500 CHF)
Falls	65,210,208	55,312,232
Pneumonia and bronchitis	13,577,547	11,516,670
Congestive heart failure	12,505,483	10,607,329
Chronic obstructive pulmonary disease	4,210,158	3,571,116
Urinary tract infection	2,695,157	2,286,070
Seizures	2,690,621	2,282,223
Diarrhea and gastroenteritis	2,077,342	1,762,032
Dehydration	700,000	593,750
Cellulitis	621,454	527,126
Hypertension	520,274	441,304
Poor glycemic control	253,310	214,862
Asthma	213,662	181,232
Total ambulatory care sensitive conditions	105,275,216	89,295,946
Other	159,449,664	135,247,488
Total	264,724,880	224,543,434

*CHF* Swiss francs

## Discussion

Hospitalizations of nursing home residents who were admitted with an ACSC were frequent and expensive for the Swiss health-care system. We observed approximately 42% of nursing home admissions due to an ACSC, half of which were attributable to falls and trauma. These findings are similar to study reports from the USA, Canada, and Sweden, where the prevalence of ACSCs excluding falls ranged between 16 and 38% (Grabowski et al. 2007; Kirsebom et al. 2014; McAndrew et al. 2015) and between 40 and 55% for studies including falls (Walker et al. 2009; Walsh et al. 2012). Our results were also similar to findings from a Swedish study documenting 25% of ED visits due to falls from nursing home residents (Kirsebom et al. 2014). In contrast, Ouslander et al. found that 67% of hospitalizations were avoidable, without considering falls, using a structured review of medical charts by experts (Ouslander et al. 2010).

Results for our cost analysis showed that admissions for ACSCs were rather costly, ranging between 105 million CHF and 89 million CHF assuming base rates of 11,200 CHF and 9500 CHF, respectively. This constitutes approximately 40% of the total costs for all nursing home admissions. The three most common ACSCs in our data—falls and trauma, pneumonia and bronchitis, and CHF—have also been reported to be among the top five conditions in other studies (Grabowski et al. 2007; McAndrew et al. 2015; Walker et al. 2009; Walsh et al. 2012; Xing et al. 2013). These common causes were also the most expensive, increasing the overall spending on ACSCs. Interestingly, dehydration, a prevalent problem in several US studies, was less prevalent in our data. This might be due to coding differences or different care practices regarding the handling of hydration in Swiss nursing homes.

We found that 40% of the costs of all hospitalizations for nursing home residents are potentially avoidable and given that the Swiss health-care system ranks above the US health-care system, this is concerning. However, it also suggests that some areas in health-care delivery could be improved. For example, falls can be prevented with measures minimizing fall risks (Walsh et al. 2012). As shown in a review, multifactorial interventions led by an interprofessional team are able to reduce both the number of fallers and recurrent fallers (Vlaeyen et al. 2015). One core problem in the management of falls in nursing homes is ruling out the diagnosis of fracture, since hardly any nursing homes have the infrastructure to run the diagnostic procedures necessary. An interesting development to counter this is mobile X-rays with telemedicine for nursing homes, which might change diagnostic procedures and reduce burdensome and costly transfers to the ED (Kjelle and Lysdahl 2017) due to the possibility of X-rays within nursing homes, reducing unnecessary ED admissions.

Staffing and skill mix is another central component to decreasing ambulatory care sensitive hospitalizations. The idea behind the development of ACSCs was that timely and effective access to ambulatory care could prevent hospitalizations (Bindman et al. 1995; Weissman et al. 1992). If residents at risk for conditions such as COPD or CHF were well monitored, some episodes of exacerbations might be avoided or detected early enough to be managed in the nursing home, although this does not apply for severe bronchospasm or hemodynamic instability for instance (Walsh et al. 2010). Similarly, early identification and treatment of pneumonia and bronchitis could prevent a hospital stay (Walsh et al. 2010). Early identification and timely intervention requires nursing home staff with the geriatric expertise able to recognize changes and act upon observations. Several studies from the USA show that the use of advanced practice nurses (APNs) reduces preventable hospitalizations (Bakerjian 2008; Kane et al. 2003; Ouslander et al. 2014; Rantz et al. 2015). These nurses with a master’s degree and training in extended clinical competencies are prepared to deliver care to vulnerable populations with complex health-care needs. In rural areas where shortages of primary care physicians are common, APNs are often the sole primary care provider for patients. Depending on US state legislature, APNs work independently or in collaboration with a physician. Within their specialty area, they assess, diagnose, and prescribe, managing the care of patients in its entirety, including referrals to physicians when indicated. In addition, they may also oversee other nursing home staff and provide training (Kane et al. 2003).

In the USA, the availability of a physician or APN for an on-site assessment of acute changes is considered a key strategy to reducing preventable hospitalizations (Ouslander et al. 2010). However, in Switzerland, access to a physician shows substantial regional variations. Patients living in regions with a high density of primary care physicians were less likely to be hospitalized due to an ACSC, whereas the risk was higher for patients living in rural areas (Berlin et al. 2014). Our results showing that more residents were admitted by ambulance for an ACSC (42%) than for non-ACSCs (31%), while it was the reverse for admission by a physician (ACSC: 46%, non-ACSC: 59%), may also reflect the issue of timely access to a primary care physician. Building a nursing home and primary care workforce with greater reliance on APNs, especially in rural areas where access to a primary care appointment may be delayed, could help reduce ACSC and associated costs in the Swiss health-care system.

Another important factor in reducing ACSCs is the implementation of advance care planning (ACP), which has been shown to increase the number of residents that die in the nursing home (Martin et al. 2016). ACP helps “ensure that people receive the medical care that is consistent with their values, goals and preferences during serious and chronic illness” (Sudore et al. 2017). This includes the appointment of a trusted person to make decisions on one’s behalf, when decision-making is no longer possible. Switzerland has recently introduced a national strategy for care planning, centered on the importance of long-term care. It outlines advance care planning and recommends discussing possible future developments and scenarios for the current illnesses, including ACSCs, to assess resident preferences and plan interventions in case of exacerbations (Bundesamt für Gesundheit und palliative ch 2018). Such care plans might also include physician orders about PRN (“as needed”) medications and documentation of wishes concerning treatment and hospitalization, which in turn supports care staff in managing acute situations when the physician is not immediately available, for example during nights and weekends.

Finally, it is important to note that programs taking a multifactorial approach have proven to be effective in reducing unnecessary hospitalizations. One such program is INTERACT, an intervention specifically designed to improve staff’s ability in early identification of complications, interprofessional communication, advance care planning, the effective treatment of chronic conditions, and timely reaction to acute changes in condition (Ouslander et al. 2014). Its effectiveness depends on the implementation of the INTERACT tools (e.g., tools to improve communication), which show the importance of the nursing homes’ motivation for change, since training and support alone will not reach the desired effect (Huckfeldt et al. 2018). Such programs are rare, and greater use should be explored to prevent hospitalizations in both the international and Swiss contexts.

The strength of the present study is the use of national data representing all Swiss hospitalizations, allowing us to provide a comprehensive description of nursing home hospitalizations. However, several limitations apply. First, the lists of conditions considered to be ambulatory care sensitive were developed in the USA and Canada and no corresponding structured expert rating was performed for the Swiss context. Although the main chronic conditions such as CHF, COPD, and pneumonia are at the forefront in ACSC lists, the inclusion of falls and trauma for nursing home residents may be controversial, since evidence for effective fall prevention is still scarce. Additionally, ACSCs provide a limited view on preventability and it is possible that not all ACSCs can be avoided in every case. In some instances, ACSCs might indicate a severe condition for which no early detection or advanced care planning could have avoided a hospitalization. Moreover, ACSCs do not take into account other system and patient level factors, such as treatment option availability in nursing homes, wishes of residents and relatives regarding hospitalizations, or residents’ overall health status. Thus, our findings on ACSCs provide valuable insight into preventable hospitalizations in Switzerland. Additional studies are needed to better understand the intricacies of different factors that favor hospitalizations.

In conclusion, hospitalizations due to ACSCs in Switzerland are frequent and costly. In order to reduce preventable hospitalizations, a multifaceted approach is needed. This should include addressing timely access to primary care with an increased use of APN roles in nursing homes; promotion of advance care planning; strengthening geriatric expertise of nursing home staff including monitoring of chronic diseases, the early detection and treatment of exacerbating symptoms, fall prevention, and the support of interprofessional communication, among other factors. The recommendation for financial reimbursement for ACP conversations at the Swiss policy level is a welcome development (Bundesamt für Gesundheit und palliative ch 2018).

## Electronic supplementary material

Below is the link to the electronic supplementary material.

Supplementary material 1 (DOCX 31 kb)
