# Supplementary material for: Preventable hospitalizations from ambulatory care sensitive conditions in nursing homes: evidence from Switzerland
Source: Int J Public Health. 2019 Sep 3;64(9):1273–81. doi: 10.1007/s00038-019-01294-1 (PMC6867979; doi:10.1007/s00038-019-01294-1)
Supplement: Supplementary file 1 — Supplementary material 1 (DOCX 31 kb) [file 38_2019_1294_MOESM1_ESM.docx]

**International Journal of Public Health**

**Preventable hospitalizations from ambulatory care sensitive conditions in nursing homes: evidence from Switzerland**

Ulrike Muench^1^, Michael Simon^2,3^, Raphaëlle-Ashley Guerbaai^2^, Carlo De Pietro^4^, Andreas Zeller^5,6^, Reto W. Kressig^5,7^, Franziska Zúñiga^2^ and the INTERCARE research group

^1^ University of California San Francisco, School of Nursing. Department of Social and Behavioural Sciences

^2^Institute of Nursing Science, Department of Public Health, Faculty of Medicine, University of Basel, Basel, Switzerland

^3^Inselspital Bern University Hospital, Nursing & Midwifery Research Unit, Bern, Switzerland

^4^Department of Business Economics, Health and Social Care at the University of Applied Sciences and Arts of Southern Switzerland, Lugano, Switzerland

^5^Faculty of Medicine, University of Basel, Basel, Switzerland

^6^Center for Primary Health Care, University of Basel, Switzerland

^7^FELIX PLATTER, University Medicine of Aging, Basel, Switzerland

**Supplementary material: ICD-10 codes to assess ambulatory care sensitive conditions**

The following table shows the ICD-10 codes used to assess the 12 ambulatory care sensitive conditions and whether they were obtained from Walker et al. (2009) or Walsh et al. (2010).

Supplementary table 1: ICD-10 codes to assess ambulatory care sensitive conditions

| **Condition** | **Walker et al. (2009)**  **ICD-9^1^** | **Walsh et al. (2010)**  **ICD-9^1^** | **Current paper**  **ICD-10^1^** |
| --- | --- | --- | --- |
| Asthma | 493 | 493.00, 493.01, 493.02 493.10, 493.11, 493.12, 493.20, 493.21, 493.22, 493.81, 493.82, 493.90, 493.91, 493.92 | J45, J450, J451, J458, J459,  J46 |
| Cellulitits | 681, | 681.00, 681.01, 681.02, 681.10, 681.11 681.9, | L03, L030, L0301, L0302, L031, L0310, L0311, L032, L033, L038, L039  L04, L040, L041, L042, L043, L048, L049  L08, L080, L088, L089  L88  L980, L983 |
|  | 682, | 682.0, 682.1, 682.2, 682.3, 682.4, 682.5, 682.6, 682.7, 682.8, 682.9, |  |
|  | 683, | 683., |  |
|  | 686 | 686.00, 686.01, 686.09, 686.1, 686.8, 686.9 |  |
| Congestive heart failure |  | 398.91 | I110, I1100, I1101  I13, I130, I1300, I1301, I131, I1310, I1311, I132, I1320, I1321  I50, I500, I5000, I5001, I501, I5011, I5012, I5013, I5014, I5019, I509  J81 |
|  | 402, | 402.11, 402.91, |  |
|  | 404, | 404.11, 404.13, 404.91, 404.93 |  |
|  | 428, | 428.0, 428.1, 428.20, 428.21, 428.22, 428.23, 428.30, 428.31, 428.32, 428.33, 428.40, 428.41, 428.42, 428.43, 428.9, |  |
|  | 518.4, | 518.4 |  |
| Seizures (Walsh)  Grand mal seizure disorders (Walker) | 345, | 345.00, 345.01, 345.10, 345.11, 345.2, 345.3, 345.40, 345.41, 345.50, 345.51, 345.60, 345.61, 345.70, 345.71, 345.80, 345.81, 345.90, 345.91, | G40, G400, G4002, G4008, G4009, G401, G402, G403, G404, G405, G406, G407, G408, G409,  G41, G410, G411, G412, G418, G419,  R56, R560, R568  (without O15, puerpural) |
|  |  | 436., |  |
|  | 780.3 | 780.31, 780.39 |  |
| Chronic-obstructive pulmonary disease | 466, | 466.0, 466.11, 466.19, | J20, J200, J201, J202, J203, J204, J205, J206, J207, J208, J209  J21, J210, J218, J219  J40,  J41, J410, J411, J418,  J42,  J43, J430, J431, J432, J438, J439,  J44, J440, J4400, J4401, J4402, J4403, J4409, J441, J4410, J4411, J4412, J4413, J4419, J448, J4480, J4481, J4482, J4483, J4489, J449, J4490, J4491, J4492, J4493, J4499,  J47 |
|  |  | 490., |  |
|  | 491, | 491.0, 491.1, 491.20, 491.21, 491.8, 491.9, |  |
|  | 492, | 492.0, 492.8, |  |
|  | 494, | 494.0, 494.1, |  |
|  | 496 | 496. |  |
| Dehydration, volume depletion (Walsh)  Dehydration (Walker) | 276.5 | 276.5,  276.8 | E86  E87.6 |
| Diarrhea and gastroenteritis (Walsh)  Gastroenteritis (Walker) | 558.9, | 558.9, | K52.9  A09, A090, A099  A02, A020, A022, A028, A029  A03, A030, A031, A032, A033, A038, A039  A05, A050, A051, A052, A053, A054, A058, A059  A060  A07, A070, A071, A072, A073, A078, A079  A04, A040, A041, A042, A043, A044, A045, A046, A048, A049  A08, A080, A081, A082, A083, A084, A085  K522, K528, |
|  | 009.0, 009.1 | 009.0, 009.1, 009.2, 009.3, |  |
|  |  | 003.0, |  |
|  |  | 004.0, 004.1, 004.2, 004.3, 004.8, 004.9, |  |
|  |  | 005.0, 005.1, 005.2, 005.3, 005.4, 005.81, 005.89, 005.9, |  |
|  |  | 006.0, |  |
|  |  | 007.0, 007.1, 007.2, 007.3, 007.4, 007.5, 007.8, 007.9, |  |
|  |  | 008.00, 008.01, 008.02, 008.03, 008.04, 008.09, 008.2, 008.3, 008.41, 008.42, 008.43, 008.44, 008.46, 008.47, 008.49, 008.5, 008.61, 008.62, 008.63, 008.64, 008.65, 008.66, 008.67, 008.69, 008.8, |  |
|  |  | 787.91 |  |
| Poor glycemic control (Walsh)  Diabetes with ketoacidosis or hyperosmolar coma (Walker)  Hypoglycemia (Walker) | 250.1–250.3 | 250.02, 250.03, | E1001, E101, E1011, E106, E1060, E1061,  E110, E1101, E111, E1111, E116, E1160, E1161,  E130, E1301, E131, E1311, E136, E1360, E1361,  E140, E1401, E141, E1411, E146, E1460, E1461,  E15,  E16.2 |
|  |  |  |  |
|  |  | 250.10, 250.11, 250.12, 250.13, 250.20, 250.21, 250.22, 250.23, 250.30, 250.31, 250.32, 250.33, |  |
|  | 251.2 | 251.0, 251.2, |  |
|  |  | 790.29 |  |
| Hypertension | 401.0, 401.9,  402.0, 402.1, 402.9 | 401.9  402.10, 402.90  403.10, 403.90  404.10 404.90 | I10, I1000, I1001, I1010, I1011, I1090, I1091  I119, I1190, I1191  I129, I1290, I1291  I131, I1310, I1311 |
| Lower respiratory: pneumonia & bronchitits (Walsh)  Pneumonia (Walker) | 481,  482.2, 482.3, 482.9,  483,  486 | 480.0, 480.1, 480.2, 480.3, 480.8, 480.9,  481.,  482.0, 482.1, 482.2, 482.30, 482.31, 482.32, 482.39, 482.40, 482.41, 482.49, 482.81, 482.82, 482.83, 482.84, 482.89, 482.9,  483.0, 483.1, 483.8,  485.,  486.  507.0 | J12, J120, J121, J122, J123, J128, J129  J13  J18, J180, J181, J182, J188, J189,  J15, J150, J151, J152, J153, J154, J155, J156, J157, J158, J159  J14  A481  J16, J160, J168  J690 |
| Kidney/urinary tract infection (Walker)  Urinary tract infection (Walsh) | 590,  599.0, 599.9 | 590.10, 590.11, 590.80, 590.81, 590.9,  595.0, 595.1, 595.2, 595.4, 595.89, 595.9,  597.0,  598.00, 598.01,  599.0,  601.0, 601.1, 601.2, 601.3, 601.4, 601.8, 601.9 | N10,  N11, N110, N111, N118, N119,  N15, N151, N1510, N1511, N158, N159,  N16, N160, N161, N162, N163, N164, N165, N168,  N291,  N30, N300, N301, N302, N303, N304, N308, N309,  N330,  N34, N340, N341, N342, N343,  N351,  N37, N370, N378,  N390# |
| Falls and trauma (Walsh)  Injuries from falls / fractures (Walker) | 800–829 or (E880–E888 *and*  800–904, 910–929, 950) | 800 – 829  830 – 839  850 – 854  905 – 909  925 – 929  940 – 949  959  991 – 992, 994 | Fracture of neck of femur (hip)  S7200, S7201, S7202, S7203, S7204, S7205, S7208, S7210, S7211, S722, S7220, S7221  Skull & face fractures  S02, S020, S0200, S0201, S0210, S021, S0211, S022, S0220, S0221, S023, S0230, S0231, S024, S0240, S0241, S025, S0250, S0251, S0260, S0261, S0262, S0264, S0265, S0266, S0268, S0269, S027, S0270, S0271, S028, S0280, S0281, S029, S0290, S0291,  T0200, T0201, T902  Fracture of upper limb  S4220, S4221, S4222, S4223, S4224, S4229, S423, S4230, S4231, S4240, S4241, S4242, S4243, S4244, S4245, S4249, S427, S4270, S4271, S428, S4280, S4281,  S52, S5200, S5201, S5202, S5209, S5210, S5211, S5212, S5219, S5220, S5221, S5230, S5231, S524, S5240, S5241, S5250, S5251, S5252, S5259, S526, S5260, S5261, S527, S5270, S5271, S528, S5280, S5281, S529, S5290, S5291,  S62, S620, S6200, S6201, S6210, S6211, S6212, S6213, S6216, S6217, S6220, S6221, S6222, S6223, S6224, S6230, S6231, S6232, S6233, S6234, S624, S6240, S6241, S6250, S6251, S6252, S6260, S6261, S6262, S6263, S627, S6270, S6271, S628, S6280, S6281,  T0220, T0221, T0240, T0241, T100, T101,  T921, T922,  Fracture of lower limb  S72, S723, S7230, S7231, S7240, S7241, S7242, S7243, S7244, S727, S7270, S7271, S728, S7280, S7281, S729, S7290, S7291,  S82, S820, S8200, S8201, S8210, S8211, S8218, S8220, S8221, S8228, S8230, S8231, S8238, S8240, S8241, S8242, S8249, S825, S8250, S8251, S826, S8260, S8261, S827, S8270, S8271, S8280, S8281, S8282, S8288, S829, S8290, S8291,  S92, S920, S9200, S9201, S921, S9210, S9211, S9220, S9221, S9222, S9223, S9228, S923, S9230, S9231, S924, S9240, S9241, S925, S9250, S9251, S927, S9270, S9271, S929, S9290, S9291,  T0230, T0231, T0250, T0251,  T120, T121,  T931, T932  Other fractures  S12, S120, S1200, S1201, S121, S1210, S1211, S1220, S1221, S1222, S1223, S1224, S1225, S127, S1270, S1271, S128, S1280, S1281, S129, S1290, S1291,  S22, S2200, S2201, S2202, S2203, S2204, S2205, S2206, S221, S2210, S2211, S222, S2220, S2221, S2230, S2231, S2232, S2240, S2241, S2242, S2243, S2244, S225, S2250, S2251, S228, S2280, S2281, S229, S2290, S2291,  S32, S3200, S3201, S3202, S3203, S3204, S3205, S321, S3210, S3211, S322, S3220, S3221, S323, S3230, S3231, S324, S3240, S3241, S325, S3250, S3251, S327, S3270, S3271, S3280, S3281, S3282, S3283, S3289,  S42, S4200, S4201, S4202, S4203, S4209, S4210, S4211, S4212, S4213, S4214, S4219, S429, S4290, S4291,  T02, T0210, T0211, T0260, T0261, T0270, T0271, T0280, T0281, T0290, T0291,  T080, T081,  T1420, T1421,  T911, T912  Intracranial injury  , S06, S060, S0600, S0601, S061, S0610, S0611, S0620, S0621, S0622, S0623, S0628, S0630, S0631, S0632, S0633, S0634, S0638, S064, S0640, S0641, S065, S0650, S0651, S066, S0660, S0661, S0670, S0671, S0679, S068, S0680, S0681, S069, S0690, S0691, T060, T90, T904, T905, T908, T909,  Crushing injury or internal injury  S04, S040, S041, S042, S043, S044, S045, S046, S047, S048, S049, S07, S070, S071, S078, S079, S090, S091, S092, S095, S097, S098, S099, S142, S143, S144, S145, S146, S15, S150, S1503, S151, S152, S153, S157, S158, S159, S16, S17, S170, S178, S179, S19, S197, S198, S199, S242, S243, S244, S245, S246, S25, S250, S251, S252, S253, S254, S255, S257, S258, S259, S26, S260, S2600, S2601, S2680, S2681, S2683, S2688, S2690, S2691, S27, S270, S2700, S2701, S271, S2710, S2711, S272, S2720, S2721, S2730, S2731, S2738, S2740, S2741, S275, S2750, S2751, S276, S2760, S2761, S2770, S2771, S2780, S2781, S2783, S2788, S2790, S2791, S28, S280, S281, S29, S290, S297, S298, S299, S342, S343, S3431, S344, S345, S346, S348, S35, S350, S351, S352, S353, S354, S355, S357, S358, S359, S36, S3600, S3601, S3602, S3603, S3604, S3608, S3610, S3611, S3612, S3613, S3614, S3615, S3616, S3618, S3620, S3621, S3630, S3631, S3640, S3641, S3649, S3650, S3651, S3652, S3653, S3654, S3659, S366, S3660, S3661, S3670, S3671, S3680, S3681, S3682, S3683, S3688, S369, S3690, S3691, S37, S3700, S3701, S3702, S3703, S371, S3710, S3711, S3720, S3721, S3722, S3728, S3730, S3731, S3733, S3738, S3740, S3741, S3750, S3751, S3760, S3761, S377, S3770, S3771, S3780, S3781, S3782, S3788, S3790, S3791, S38, S380, S381, S39, S390, S396, S397, S398, S3980, S3988, S399, S44, S440, S441, S442, S443, S444, S445, S447, S448, S449, S45, S450, S451, S452, S453, S457, S458, S459, S46, S460, S461, S462, S463, S467, S468, S469, S47, S49, S497, S498, S499, S54, S540, S541, S542, S543, S547, S548, S549, S55, S550, S551, S552, S557, S558, S559, S56, S560, S561, S562, S563, S564, S565, S567, S568, S57, S570, S578, S579, S59, S597, S598, S599, S64, S640, S641, S642, S643, S644, S647, S648, S649, S65, S650, S651, S652, S653, S654, S655, S657, S658, S659, S66, S660, S661, S662, S663, S664, S665, S666, S667, S668, S669, S67, S670, S678, S69, S697, S698, S699, S74, S740, S741, S742, S747, S748, S749, S75, S750, S751, S752, S757, S758, S759, S76, S760, S761, S762, S763, S764, S767, S77, S770, S771, S772, S79, S797, S798, S799, S84, S840, S841, S842, S847, S848, S849, S85, S850, S851, S852, S853, S854, S855, S857, S858, S859, S86, S860, S861, S862, S863, S867, S868, S869, S87, S870, S878, S89, S897, S898, S899, S94, S940, S941, S942, S943, S947, S948, S949, S95, S950, S951, S952, S957, S958, S959, S96, S960, S961, S962, S967, S968, S969, S97, S970, S971, S978, S99, S997, S998, S999, T04, T040, T041, T042, T043, T044, T047, T048, T049, T062, T063, T064, T065, T094, T147, T903, T914, T915, T918, T919, T92, T924, T925, T926, T928, T929, T93, T934, T935, T936, T938,  Open wounds of head, neck, & trunk  S01, S010, S011, S012, S0120, S0121, S0123, S013, S0130, S0131, S014, S0141, S0143, S0149, S015, S0151, S0153, S0154, S0155, S0159, S017, S018, S0180, S019, S052, S053, S054, S055, S056, S057, S058, S08, S080, S081, S088, S089, S11, S110, S1102, S111, S112, S117, S118, S1180, S119, S18, S21, S210, S211, S212, S217, S218, S2180, S219, S31, S310, S311, S312, S313, S314, S315, S317, S318, S382, S383, S41, S410, S417, S418, S4180, S48, S480, S481, S489, S71, S710, S717, S718, S7180, S78, S780, T01, T010, T011, T018, T019, T05, T058, T059, T091, T096, T901,  Open wounds of extremities  S411, S51, S510, S517, S518, S5180, S519, S58, S580, S581, S589, S61, S610, S611, S617, S618, S6180, S619, S68, S680, S681, S682, S683, S684, S688, S689, S711, S781, S789, S81, S810, S817, S818, S8180, S819, S88, S880, S881, S889, S91, S910, S911, S912, S913, S917, S9180, S98, S980, S981, S982, S983, S984, T012, T013, T016, T050, T051, T052, T053, T054, T055, T056, T111, T116, T131, T136, T920, T930  Superficial injury, contusion  S00, S000, S0000, S0001, S0005, S0008, S001, S002, S0021, S003, S0031, S0035, S004, S0045, S005, S0051, S0052, S0053, S0055, S007, S008, S0080, S0081, S0084, S0085, S0088, S009, S0090, S0091, S0095, S05, S050, S051, S059, S10, S100, S101, S1010, S1013, S1018, S107, S108, S1085, S109, S20, S200, S201, S2018, S202, S203, S2038, S204, S2041, S2048, S207, S208, S2081, S2085, S30, S300, S301, S302, S307, S308, S3080, S3085, S3088, S309, S3091, S3095, S40, S400, S407, S408, S4081, S4088, S409, S50, S500, S501, S507, S508, S5081, S5088, S509, S60, S600, S601, S602, S607, S608, S6081, S6083, S6084, S6088, S609, S70, S700, S701, S707, S708, S7081, S7084, S7088, S709, S80, S800, S801, S807, S808, S8081, S8082, S8083, S8088, S809, S90, S900, S901, S902, S903, S907, S908, S9081, S9082, S9084, S9088, S909, T00, T000, T001, T002, T003, T006, T008, T009, T090, T0905, T110, T1105, T130, T1300, T1305, T900, T91, T910,  Burns  T20, T200, T201, T202, T2020, T2021, T203, T204, T205, T206, T207, T21, T210, T211, T212, T2121, T2122, T2123, T2124, T213, T2131, T2132, T2133, T2134, T2139, T214, T215, T216, T217, T2182, T2183, T2184, T2190, T22, T220, T221, T222, T2220, T2221, T2222, T223, T2230, T2231, T2232, T2233, T224, T225, T226, T227, T2271, T2281, T2282, T23, T230, T231, T232, T2320, T2321, T233, T234, T235, T236, T237, T24, T240, T241, T242, T2420, T2421, T243, T244, T245, T246, T247, T25, T250, T251, T252, T2520, T2521, T253, T254, T255, T256, T257, T26, T260, T261, T262, T263, T264, T265, T266, T267, T268, T269, T27, T270, T271, T272, T273, T274, T275, T276, T277, T28, T280, T281, T282, T283, T284, T285, T286, T287, T288, T289, T29, T290, T291, T292, T293, T294, T295, T296, T297, T30, T300, T301, T302, T303, T304, T305, T306, T307, T31, T310, T311, T312, T313, T314, T315, T316, T317, T318, T319, T32, T320, T321, T322, T323, T324, T325, T326, T327, T328, T329, T95, T950, T951, T952, T953, T954, T958, T959  Other injuries & conditions due to external causes  R090, R785, R786, T06, T068, T07, T09, T095, T098, T099, T11, T113, T114, T115, T118, T119, T13, T133, T134, T135, T138, T139, T14, T1400, T1402, T1403, T1404, T1405, T1408, T141, T144, T145, T146, T148, T149, T15, T150, T151, T158, T159, T16, T17, T170, T171, T172, T173, T174, T175, T178, T179, T18, T180, T181, T182, T183, T184, T185, T188, T189, T19, T190, T191, T192, T193, T198, T199, T33, T330, T331, T332, T333, T334, T335, T336, T337, T338, T339, T34, T340, T341, T342, T343, T344, T345, T346, T347, T348, T349, T35, T350, T351, T352, T353, T354, T355, T356, T357, T66, T67, T670, T671, T672, T673, T674, T675, T676, T677, T678, T679, T68, T69, T690, T691, T698, T699, T70, T700, T701, T702, T703, T704, T708, T709, T71, T73, T730, T731, T732, T733, T738, T739, T74, T740, T741, T742, T743, T748, T749, T75, T750, T751, T752, T753, T754, T758, T78, T788, T789, T79, T790, T791, T792, T793, T794, T795, T796, T797, T798, T799, T939, T94, T940, T941, T98, T980, T981, T982 |
| **Other diagnoses not used in current paper:** |  |  |  |
| Angina pectoris | 411.1, 411.8, 413 |  |  |
| Dental conditions | 521–523, 525, 528 |  |  |
| Diabetes with specified manifestations | 250.8, 250.9 |  |  |
| Diabetes without specified complications | 250.0 |  |  |
| Septicemia | 0031, 0223, 038, 0545 |  |  |
| Altered mental status / acute confusion / delirium |  | 290.3, 290.41,  292.81,  293.0, 293.1 |  |
| Anemia |  | 280.0, 280.1, 280.8, 280.9,  281.0, 281.1, 281.2, 281.3, 281.4, 281.8, 281.9,  285.21, 285.22, 285.29, 285.9 |  |
| Hypotension |  | 458.0, 458.1, 458.21, 458.29, 458.8, 458.9 |  |
| Hyponatremia |  | 276.1 |  |
| Acute renal failure |  | 584.5, 584.6, 584.7, 584.8, 584.9,  588.81, 588.89, 588.9 |  |
| Constipation / fecal impaction / obstipation |  | 560.39,  564.00, 564.01, 564.09 |  |
| Clostridium Difficile |  | 008.45 | A047 |
| Skin ulcers |  | 707.00, 707.01, 707.02, 707.03, 707.04, 707.05, 707.06, 707.07, 707.09, 707.10, 707.11, 707.12, 707.13, 707.14 707.15, 707.19, 707.8, 707.9 |  |
| Psychosis, severe agitation, organic brain syndrome |  | 290.42, 290.43, 290.8, 290.9,  293.81, 293.82, 293.83, 293.84, 293.89, 293.9,  297.0, 297.1, 297.2, 297.3, 297.8, 297.9  298.0, 298.1, 298.2, 298.3, 298.4, 298.8, 298.9 |  |
| Failure to thrive – weight loss |  | 783.21, 783.22, 783.3, 783.7 |  |
| Nutritional deficiencies |  | 260.,  261.,  262.,  263.0, 263.1, 263.2, 263.8, 263.9,  268.0, 268.1 |  |

^1^ ICD: International Statistical Classification of Diseases and Related Health Problems
